# Simbu Viruses’ Infection of Livestock in Israel—A Transient Climatic Land

**DOI:** 10.3390/v13112149

**Published:** 2021-10-25

**Authors:** Jacob Brenner, Adi Behar

**Affiliations:** Department of Parasitology, Kimron Veterinary Institute, Rishon LeZion 50250, Israel; brennerjacovet@gmail.com

**Keywords:** *Orthobunyavirus*, *Culicoides*, emerging disease, Mediterranean basin, Simbu viruses

## Abstract

Important lessons have been learned by the Israeli veterinary community regarding Simbu serogroup viruses infections. This serogroup of viruses might cause the births of neonatal malformation in susceptible ruminant’s populations. Until 2012, only Akabane virus was connected with the births of malformed ruminants in Israel. However, serological and genomic detection tests, coupled with viral isolations, revealed that more than a single Simbu serogroup serotype could be present concurrently in the same farm or even in the same animal. From 2012 to date, Aino, Shuni, Shamunda, Satuperi, Peaton, Schmallenberg, and Sango viruses have been found in Israel either by serological or genomic investigation. Israel is located in the Eastern Mediterranean Basin, a terrestrial and climatic bridge between the three old continents. The Eastern Mediterranean shores benefit from both the tropical/subtropical and the continental climatic conditions. Therefore, the Eastern Mediterranean basin might serve as an optimal investigatory compound for several arboviral diseases, acting as a sentinel. This review summarizes updated information related to the presence of Simbu serogroup viruses in Israel.

## 1. Introduction

Simbu serogroup of viruses is one of the largest serogroups within the genus *Orthobunyavirus* of the family *Peribunyaviridae*, comprises at least 25 antigenically different but serologically related viruses. Similar to all members of the genus *Orthobunyavirus,* Simbu serogroup viruses have a tripartite, negative sense (−) single-stranded (ss) RNA genome composed of L (large), M (medium) and S (small) segments. These viruses are transmitted mainly by *Culicoides* biting midges, and they persist in the environment by cycling between infected mammalian hosts and *Culicoides* vectors. Some Simbu serogroup viruses are responsible for outbreaks of congenitally deformed ruminant neonates; the arthrogryposis-hydranencephaly syndrome (AH-S) [1,2]. In infected adult ruminants, abortions, dystocia, fever and reduced milk production [3,4] and neurological and rabies-like manifestations might be observed [5,6,7] (. The group at risk of infection is pregnant females. Several viruses from this serogroup have been shown to cross the placenta of ruminants to the developing fetus: Akabane (AKA), Satuperi (SAT), Aino (AINO), Shamonda (SHA), Shuni (SHU), Peaton (PEA) and Schmallenberg (SB) viruses. Replication of these viruses in the developing fetus can cause outbreaks of abortion, stillbirth and malformations, seen only at birth [1,2,3,6,8,9,10,11,12,13,14,15,16,17,18,19] The neonatal skeletal malformations are known as arthrogryposis. The central neurological damages can range from microscopic to mild or severe and includes hydranencephaly, microencephaly, and polio-encephalomyelitis. The brain damage is correlated with the stage of pregnancy at which the mother is infected. For instance, severe brain malformations in cattle may occur if the naïve female is infected between 76 and 106 days of pregnancy [2]. Oropouche virus from South America [20], Cache Valley virus from North America [21], and SHUV are implicated in clinical manifestations in humans. They are connected with viruses–associated–aseptic–meningoencephalitis [22]. The Simbu serogroup given names suggests their primary places of origin. Most of them have been firstly found in the tropical-subtropical zones (For example, Simbu is an area in Kivu, Democratic Republic of the Congo) that are endemically swarming with blood-sucking insects. These insects can serve as vectors for viruses that might cause infectious diseases around the globe when and where they encounter naïve stocks of animals.

**Figure 1:**
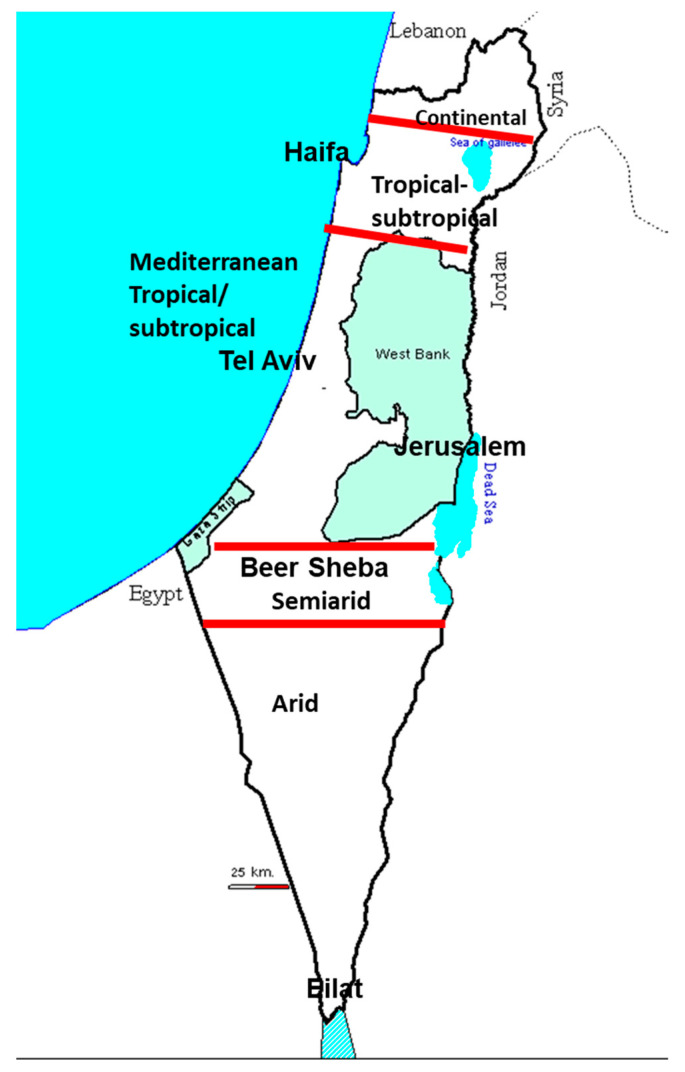
Schematic demonstration of the various climatic zones in Israel.

This review aims to summarize the findings of the Simbu serogroup viruses’ presence in Israel to date. AH-S outbreaks related solely to AKAV infections were documented in 1969–1970, 2001–2002 and 2012 [8,9,13,18,23,24]. Additional AH-S vast outbreaks erupted in 2014/15, and 2018/19 were connected with infections of SHUV and SBV, respectively [5,10,16,19,25]. Further studies revealed the presence of SATV, SHAV, PEAV, and Sango (SV) [17,18,26,27]. Additionally, studies revealed that several Simbu serogroup serotypes are concurrently found in the same farm or animal [26]; At least one member of the Simbu serogroup virus might infect animals every year [18] and that the infected *Culicoides* spp. (i.e., *C. imicola, C. oxystoma* and *C. puncticulis*) circulates all year long due to Israel’s tropical and continental climatic conditions (Figure 1) [18]. 

These epidemiological findings suggest that Israel may provide an idyllic environment for distinct serotypes of Simbu serogroup viruses to exchange genome segments by ‘reassortment’ within the vertebrate (ruminant) or invertebrate hosts (*Culicoides* midge). Thus, allowing these viruses to evolve and adapt to local conditions and ecosystems rapidly. Consequently, more dangerous biological properties of these viruses such as persistence, virulence and distribution are likely to occur and emerge within both vector and host populations in the Israeli farms, making Israel an optimal investigatory compound for these viruses.

## 2. First Arthrogryposis-Hydranencephaly Syndrome Outbreak in Israel (1969/1970) Attributed to AKAV Infections

An outbreak of congenital malformations in ruminant farm populations characterized by AH-S appeared in Israel in 1969. Based on serological, epidemiological, clinical, pathological and histopathological evidence, the causal agent was diagnosed as AKAV. To corroborate the possible links between AKAV infection of pregnant dams and the appearances of the AH-S in newborns ruminants, experimental inoculation of pregnant cows with AKAV was carried out. The outcomes confirmed the existence of the link between AKAV infection of pregnant cows and the birth of deformed calves in Israel and elsewhere [8,9,10,11,12,13] (Table 1).

## 3. Clinical Findings in the 2001–2002 AKAV Infections 

**Figure 2:**
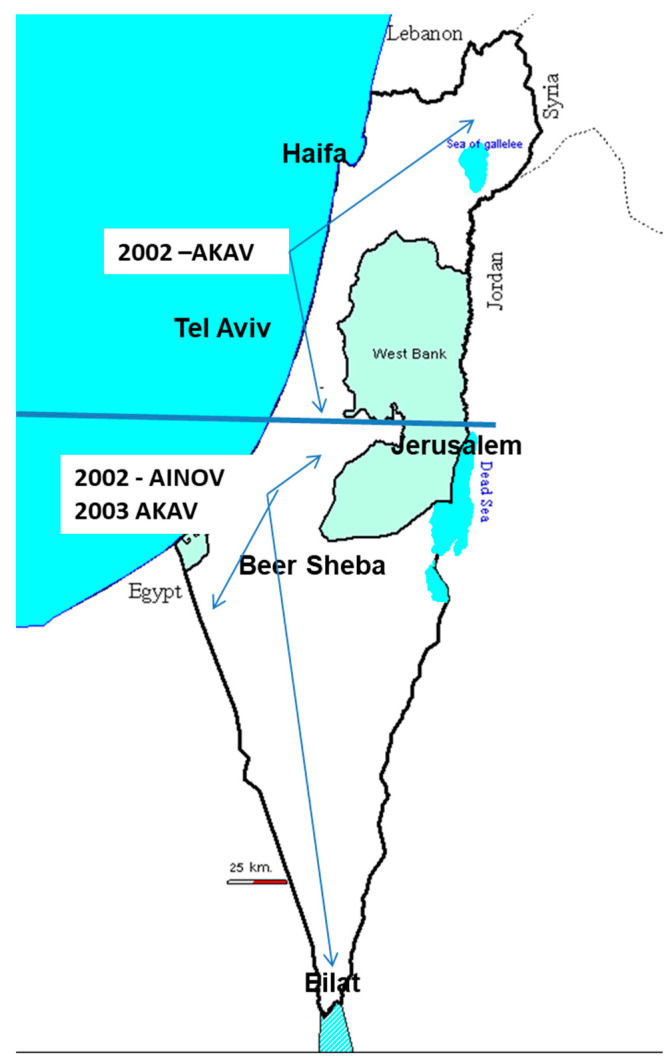
This figure describes the dichotomy of arthrogryposis hydranencephaly syndrome appearances in 2002 due to the Akabane virus attack in 2001 in the northern regions. The sequels of the second attack were observed in 2003 in the southern regions only as the virus spread southward during 2002 (the counter expected direction). AKAV, AINOV: Akabane and Aino virus, respectively.

In February 2002, the first cases of blind newborn calves (one of the most characteristic pathological features of AH-S) appeared in the northern regions of Israel [14]. The new clinical cases were reported by field practitioners, and all of them occurred in the above latitude 31°00′. Since the disease is mainly seasonal, covering the Israeli winter and early spring, it was postulated that the malformations seen in 2002 resulted from viral attacks that had probably occurred in the late summer-early autumn of 2001. Simbu serogroup viruses were the suspected etiological agents responsible for the 2002 outbreak of blind newborn calves, similar to what had occurred with AKAV in the 1969–1970 outbreak [8,9,10,11,12,13] (Figure 2). The primary epidemiological tool available at that time was to conduct a case-control study. Therefore, Simbu virus seroprevalence and seroreactivity were compared between affected ruminant herds above latitude 31°00′ (target group) vs. those located below that latitude (control group). Sera collected during the outbreak were submitted for specific virus-neutralization tests (VNTs) against a panel of selected Simbu serogroup viruses. The clear-cut sero-neutralizing outcome indicated that AKAV was the sole attributive infective agent of the 2002 AH-S outbreak. However, an additional virus was found to be circulating in our region—AINOV [14,15] (Figure 2). AH-S reappeared again between February and May 2003 in the south of Israel below latitude 31°00′. The malformations in the south were thought to be the consequence of the same epizootic virus as in 2002 that had spread during the 2001 AKAV outbreak [14,15]. It seems that the ruminant population remained serologically unprotected and therefore suffered a subsequent AKAV attack that infected the naïve population housed with the southern dairy cattle herds.

In 2001, a novel polymerase chain reaction (PCR) procedure that allowed the detection of AKAV from affected cattle brains and *Culicoides imicola* was developed [23,25]. This novel PCR assay, combined with serum samples and pre-colostral sera from clinical cases, pointed directly to AKAV as the agent responsible for the 2002 blind newborn calf syndrome. It should be noted that the Israeli AKAV strain became a new distinct lineage [25]. 

## 4. Clinical Findings in the 2011 AKAV Infection 

**Figure 3:**
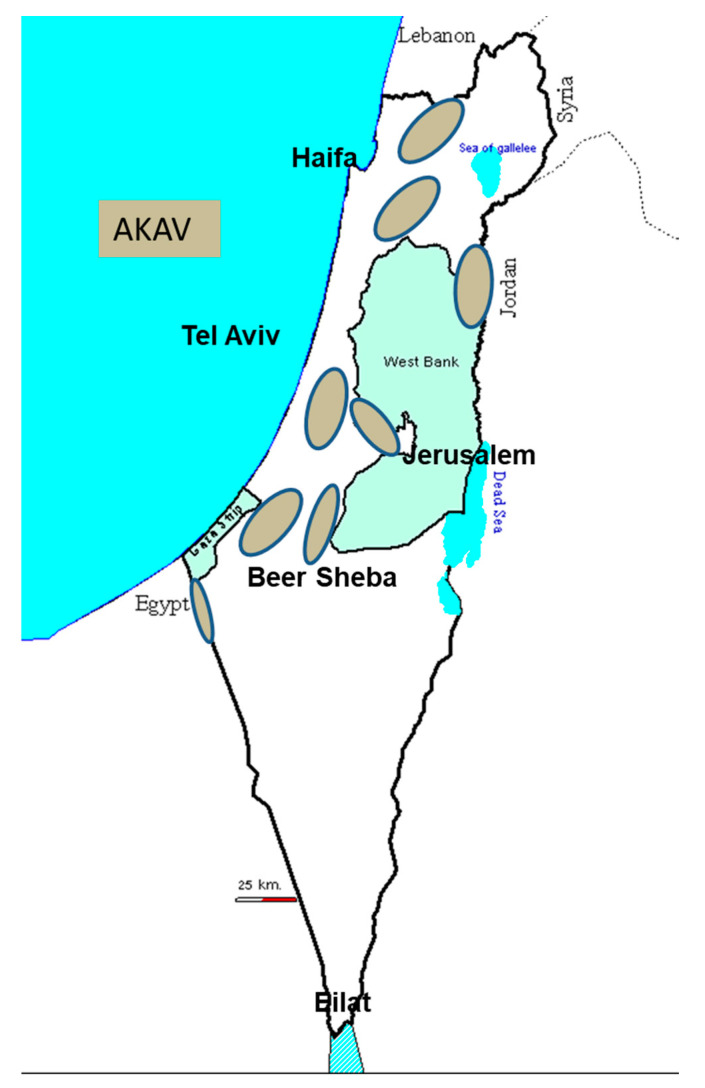
This figure portrays the arthrogryposis hydranencephaly syndrome reported in 2012 linked to Akabane virus (AKAV) infections in 2011.

The 2011 AKAV infection was associated with the appearance of AH-S syndrome in 2012. It was assessed using the same specific PCR protocol developed during the 2002–2003 AKAV attacks on the Israeli ruminant populations [23,25]. AKAV detection was carried out on samples derived from suspected pathological biopsies and whole non-coagulated blood samples. The aim was to assess which of the Simbu serogroup viruses was responsible for the births of calves presenting AH-S at the beginning of the winter of 2012. No serological tests were performed, but each suspected sample was analyzed by the AKAV-RNA-specific PCR procedure (Figure 3) [5,25,26]. 

Most of the suspected samples were collected with a focus on the Central Coastal Plain. When available, the Kimron Veterinary Institute (KVI) team collected pairs of samples, namely, from the malformed newborn calves and their respective dams (7 pairs for 8 neonates). Specific AKAV-RNA fragments were detected in all of the suspected samples. In addition, two adult lactating dams that had given birth to malformed calves were tested, and AKAV was detected in their brains. Interestingly, specific AKAV-RNA fragments were detected only from samples taken from the hippocampus—no such fragments were found in other brain tissues [5]. In parallel, 40 unclothed blood samples were collected in the field or taken from the stored sera of abortive material at the KVI. The samples were associated with reproductive disorders. These samples represented more than a hundred herds scattered along all Israeli regions which have not been suspected as being affected by the disease. All the tested sera were negative to other known abortive pathogens.

Other samples came from the brain tissue of aborted malformed fetuses (n = 12) and adult cows (n = 16) with central nervous system manifestations that had been sent to the KVI for rabies diagnosis. Half of the 40 tested serum samples were positive for AKAV RNA, as were 6 of 16 brain samples from adult cows tested by nested PCR [25,26]. 

## 5. Seroreactivity Findings Obtained during an Active Sero-Surveillance Period from 2008–2014 

In 2014, a serosurvey study was conducted to find the etiology of the seroconversion in selected, presumably naïve heifers at the time of blood sampling (June/July-2014; time 0), where applicable. In addition, those samples were used to determine to which Simbu serogroup viruses the adult cow populations had been exposed to in the past by analyzing their seroreactivity toward a panel of selected Simbu serogroup viruses [26]. For this study, farms from 5 regions in Israel have been selected: three different valleys in Northern Israel, the Southern Coastal Plain and the Central Coastal Plain.

**Figure 4:**
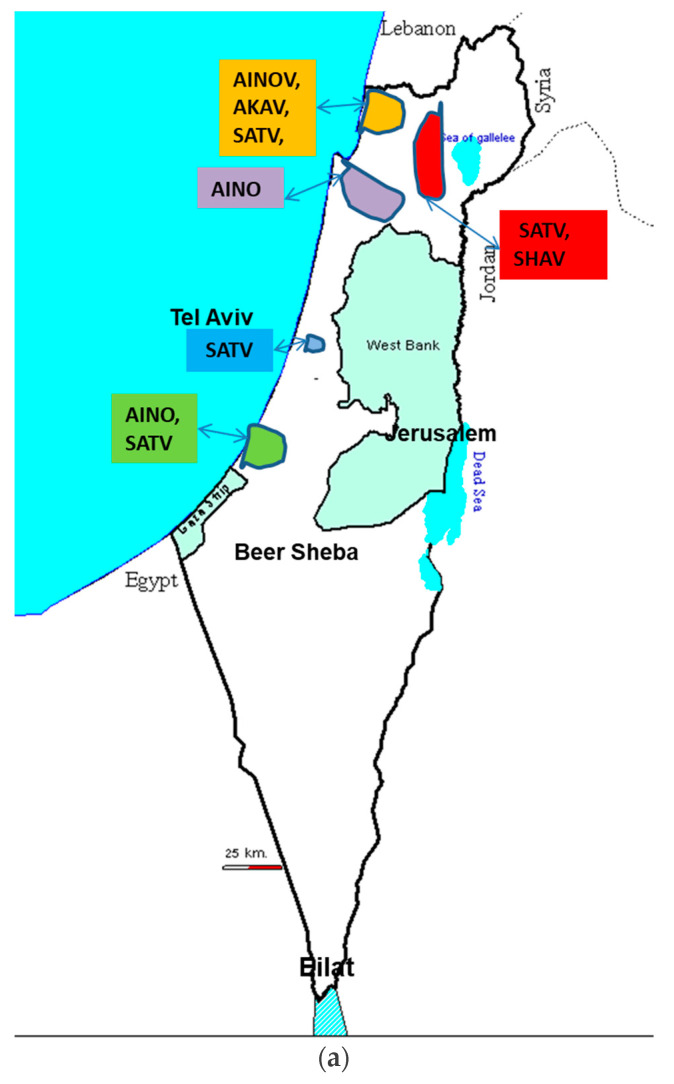

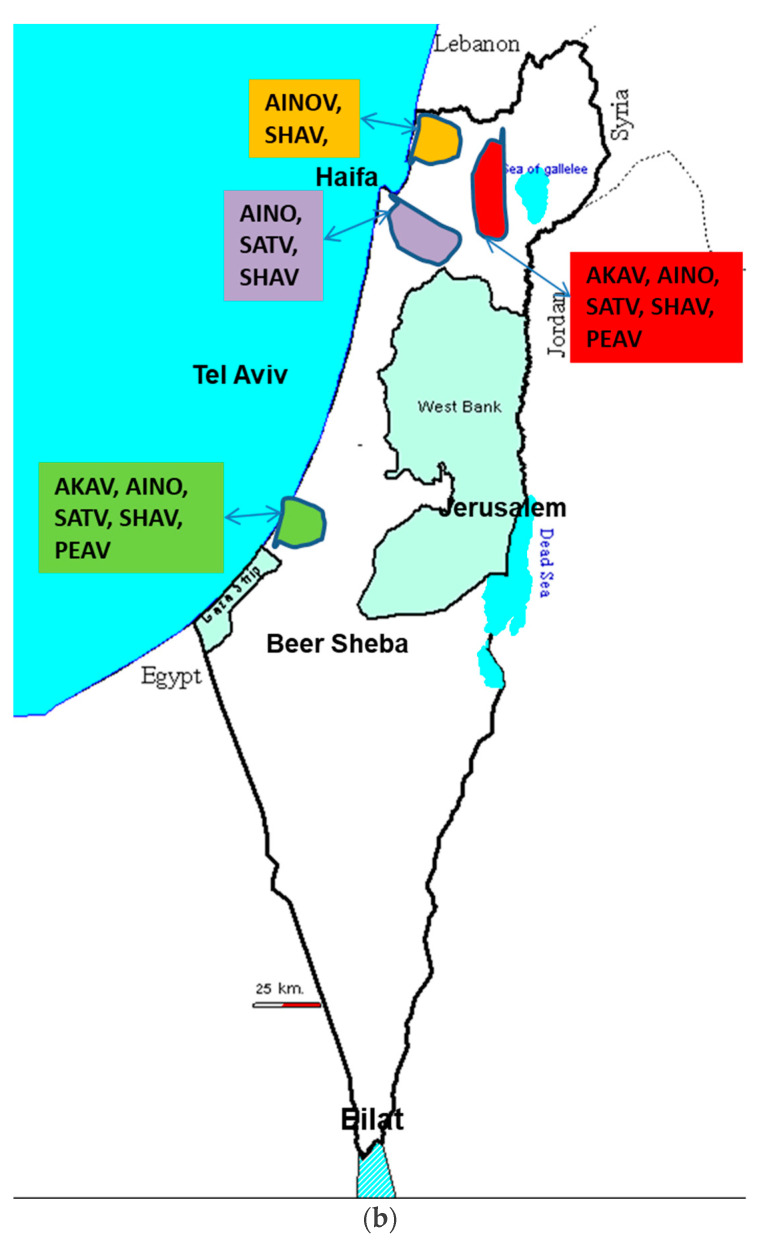
These figures show the anamnestic Simbu viruses reactive sera of adult milking cows born before 2010 (**a**) and heifers seroconverted in 2013 summer-early autumn (**b**). AKA, AINO, PEA, SAT, SHAV, SHU: Akabane, Aino, Peaton, Satuperi, Shamunda, Shuni, viruses.

The serum samples found positive by ELISA were further positive by virus-neutralizing tests to AKAV, AINOV, SATV, SHAV, and PEAV. Antibody detection in lactating adult cows revealed that several viruses were circulating in Israel between 2008 and 2014 (Figure 4a) and that the same herd was exposed to several different Simbu serogroup viruses concomitantly. Antibody detection in heifers indicated that the ruminants became seropositive after being exposed to more than one Simbu serogroup virus concurrently during the autumn of 2014 (Figure 4b). It should be mentioned that several ELISA reactive sera resulted negative when tested by VNT against the selected Simbu viruses suggesting the presence of yet unidentified virus in our region [26].

## 6. Findings during the 2014/2015 and 2018/2019 SHUV Outbreak 

**Figure 5:**
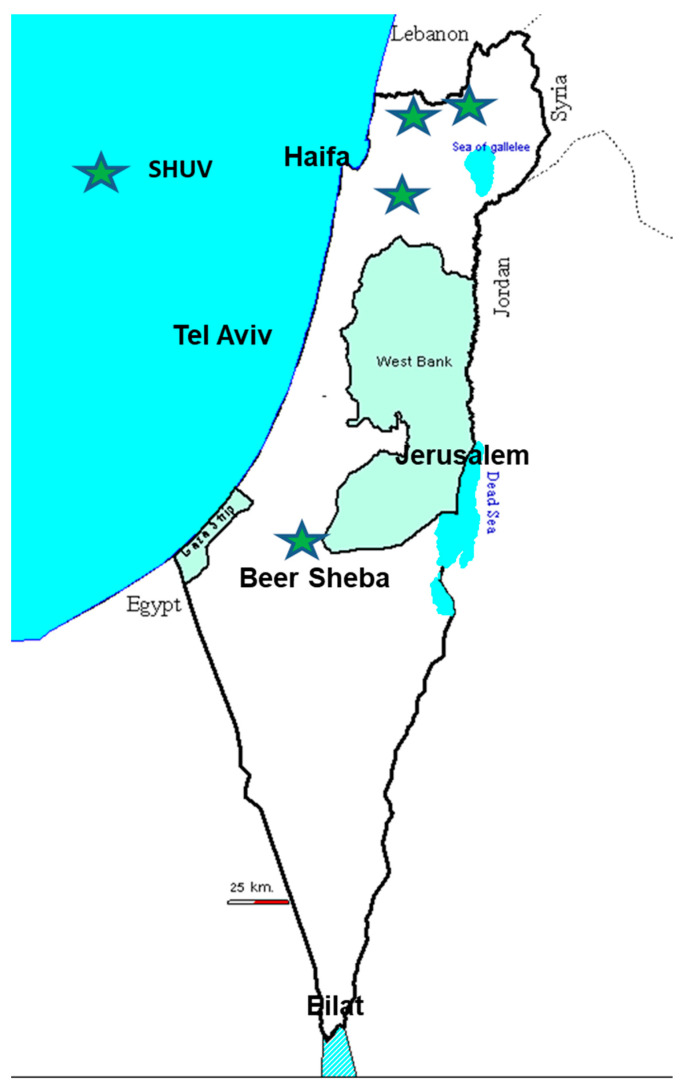
This figure shows the infected sites of arthrogryposis hydranencephaly syndrome associated with Shuni-virus (SHUV) in 2014/2015 and 2018/2019.

In December 2014, SHUV was isolated for the first time in Israel. This first case originated from a malformed lamb belonging to a herd that had exhibited multiple cases of neonatal malformation and decreased progeny prolificacy. Additional samples from 15 clinical cases from cattle, sheep and goats were also SHUV PCR positive. Most of the reported and confirmed clinical cases were from the northern Israeli valleys, but one was from a sheep housed in a nomadic herd in the north marginal of the Negev Desert [6,16]) (Figure 5). Interestingly, both SHUV elicited cerebral-neurological manifestations, rabies-like, in adult and parturient cows in Israel similar to those reported in 2012 that were linked to AKAV infection [5,6,16]. 

## 7. Genomic Detection of Additional Simbu Serogroup Viruses between 2015 and 2017 

**Figure 6:**
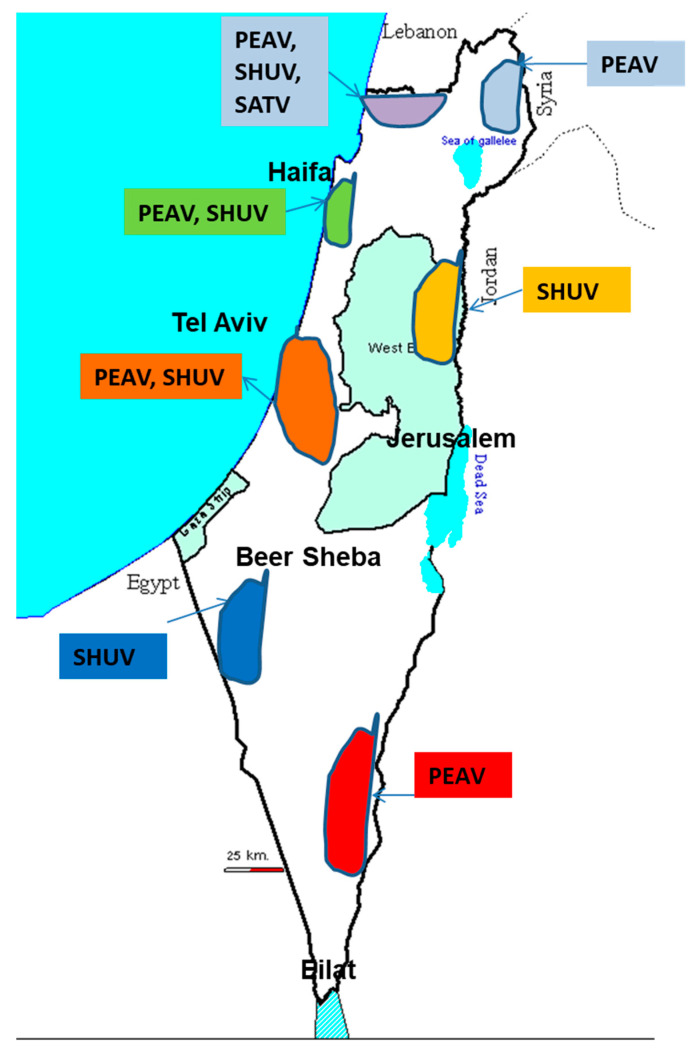
This figure shows the Simbu infected sites detected by genomic tests between 2016 and 2019. PEA, SHU, SAT: Peaton, Shuni, Satuperi viruses, respectively. (Schmallenberg virus is not shown. See Figure 7).

After the SHUV outbreak, an arboviral monitoring system was established at KVI. Blood samples and *Culicoides* biting midges were collected every month from 11 selected dairy farms representing eight different geographical regions in Israel [17,18,19]. The monitoring system was able to detect seroconversion to Simbu serogroup viruses each year from 2015 to 2019 (Figure 6). In 2017, RNA fragments of PEAV were detected in the cerebral spinal fluid (CSF) of a malformed calf with hydranencephaly from one of the cattle farms selected for the monitoring system [17]. The calf was born in March 2017 to a heifer located in a dairy cattle farm in the Central Coastal Plain that seroconverted in July 2016. The systematic arbo monitoring project also yielded the detection of PEAV, SHUV and SATV RNA fragments from pools of *C. imicola*, *C. oxystoma*, *C.* *puncticollis* from 2015 to 2017 [17,18]. 

## 8. Schmallenberg Virus Outbreak 2018–2019 

In 2018 and 2019, few ruminants’ herds exhibited AH-S in which genomic detections of Schmallenberg Virus (SBV) were confirmed. Moreover, specific genomic fragments of SBV were detected in *C. imicola*, *C. oxystoma*, *C.* *puncticollis* and *C. newsteadii* pools collected as part of the systematic arbo monitoring project and in specific collections in the vicinity of the affected premises. Infected *Culicoides* have been captured from the Golan Highest, near the Syrian and Lebanese borders, to the far south of the Negev desert, indicating this virus’s spread. Even though being searched for a decade, SBV has not been detected in Israel before [19]. It should be noted that considering the numbered of clinically reported SBV affected herds, its potential pathogenicity seemed weaker than the AKAV ones. It should be emphasized that SBV appears to be a reassortant, deriving the M RNA segment from SATV and the S and L RNA segments from SHAV probably due to coinfection of these viruses either in *Culicoides* vectors or in the ruminant hosts [28]. 

## 9. Discussion

Before 2000, arbovirus infections attracted little or no attention from Western practitioners, especially in Europe. Essentially, orbivirus infections were ignored and regarded as not posing a threat to the European livestock industry. However, bluetongue disease forced European scientists to rethink this assumption around the turn of the millennium [29]. Further evidence for the presence of arboviruses in Europe came with the detection of SBV [3]. 

An illustrative example of the strategies employed in Israel to cope with emerging diseases, especially those that have reemerged with cycles every several years, is the response to cyclic AH-S epidemics connected to AKAV infections. The causative agent was related to the first A-H syndrome diagnosed in 1969–1970, approximately four years after the clinical features of the epidemic had been described [8,9,10,11,12,13]. Contrary, in the 2001–2002 AH-S outbreaks, after already familiar with the previous outbreak’s clinical manifestations, the veterinary community raised the suspicion of possible Simbu serogroup virus involvement sooner [14,15]. Indeed, the results were available within only a few months of the appearance of the AH-S by using the VNT comparing samples from affected versus unaffected regions. However, samples from the unaffected areas revealed the anamnestic circulation of an additional Simbu virus, AINOV, suggesting that AKAV was not the only Simbu virus in this region [14]. Notably, in VNTs, there is strong cross-reactivity between AINOV and SHUV [1,2]. (Since the 2002 outbreak, AINOV has only been detected by VNT. Thus, the possibility that this was the entrance point of SHUV into the Middle East cannot be ruled out as serology is not specific enough. Indeed, the 2012 AH-S outbreak, also connected with an AKAV infection, was diagnosed within a few weeks of receiving infected animal samples [5], using specific AKAV PCR procedures developed following the 2001–2002 outbreak [23,25]. 

In 2012 SBV was identified in Europe [3]. This finding raises several questions. How was the Middle East spared from SBV? Where does SBV come from? Could other Simbu serogroup viruses be circulating in Israel or Europe [4]?

To clarify some of these questions, a serosurvey aimed at identifying additional Simbu serogroup viruses present in Israel was designed. The VNT results revealed the presence of SATV, SHAV and PEAV in Israel [26]. Consolidating the claim that Simbu serogroup viruses have invaded the region, SHUV, PEAV, SHAV, and SV genomic fragments were detected in pathological animals’ material and/or in *Culicoides* collected between 2015 and 2021 [17,18,27]. 

Epidemiological studies conducted from 2015 to 2019 confirmed the entrance of SBV to Israel in 2018 [19]. This Simbu virus has been regarded as an elusive AH-S agent in the Eastern Mediterranean Basin, although massively present in Europe and the Southern Mediterranean coasts [3]. This phenomenon contradicts the generally accepted arboviruses spreading from Africa to Europe without leaving naïve pockets in-betweens.

A systematic insect monitoring offered more data regarding the different geographical regions of Israel. The surveillance mentioned above showed that various *Culicoides* spp., carry Simbu serogroup viruses. Specific Simbu genomic material was detected not only in *C. imicola* but also in *C. oxystoma*, *C. puncticulis*, and *C. newsteadii* [17,18,19]. Given that our knowledge of arboviruses and their vectors is lacking, we fully expect the detection of more arboviruses in future monitoring. Recent detection and isolation of SBV and SV [19,27] might suggest that our assumption is probably correct.

Located in the eastern Mediterranean basin, Israel is a crossroads between the three old continents and benefit from tropical and continental climatic conditions. Recent studies have shown that a large diversity of Simbu serogroup viruses representing Asia and the east (AKAV, AINOV, PEAV); Africa (SHUV, SHAV, STAV, SV) and Europe (SBV) are present in it. Thus, Israel might serve as an optimal location for comparative studies on these viruses.

For example, the combination of these environmental conditions provides an idyllic setting for distinct serotypes to exchange genome segments within the vertebrate (ruminant) or invertebrate (*Culicoides*) hosts by process of ‘reassortment’.

Finally, most of the known Simbu serogroup viruses originate where investigative scientific organizations are weak. Therefore, to predict their routes of spreading seems quite impossible so that, their complex and tortuous epidemiology can only be suggestive but not conclusive. The well organized, industrialized countries, mainly in Europe, need to establish laboratories that are well equipped to rapidly deal with the intrusion of different members of these viruses into their territories and protect themselves from surprises.

## Figures and Tables

**Figure 7:**
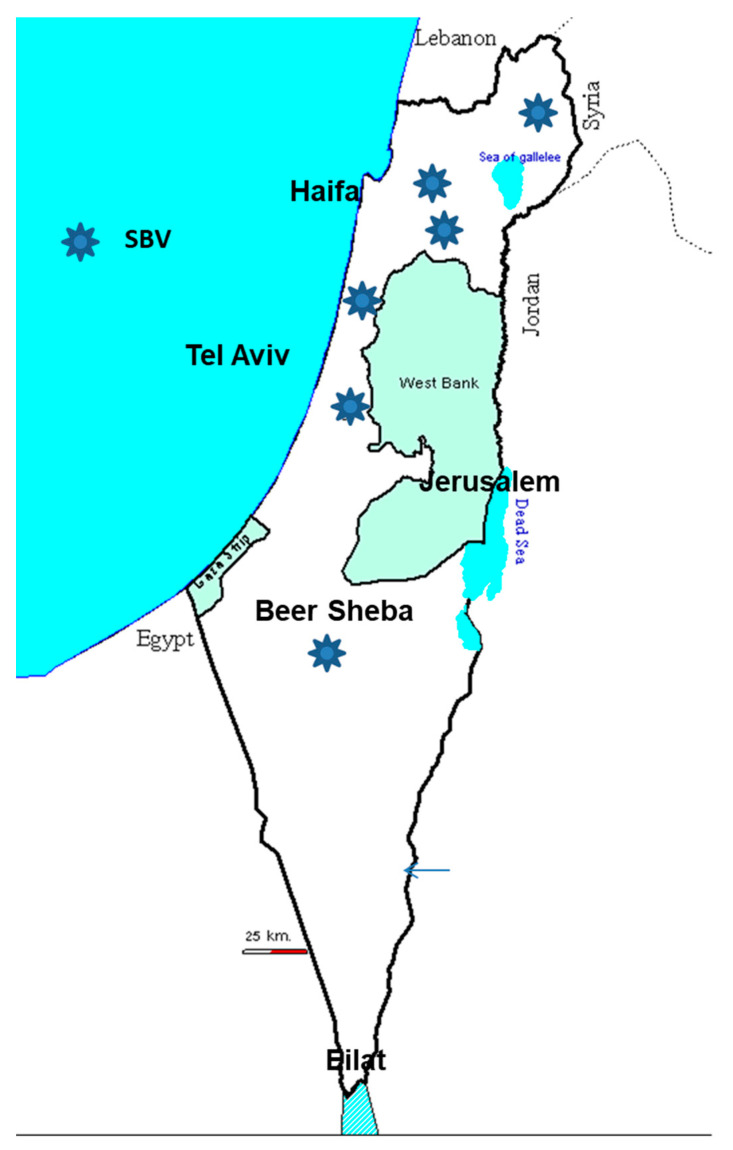
This figure shows the Schmallenberg virus (SBV) infected sites detected from 2018 to date.

**Table 1:** Significant clinical and epidemiological tools demonstrate the presence of Simbu serogroup viruses in Israel (2000–2020).

Outbreak	Identified Agent	Major Clinical Aspects	Epidemiological and Laboratory Approaches
2001–2003	AKAV, AINOV	Blind newborn calf syndrome hydranencephaly syndromes Detection in *C. imicola*	Specific serological VNTs comparison of clinically affected and unaffected zones [14]. RT-qPCR [25].
2008–2012	AKAV, AINO, PEAV, SATV, SHAV	Not relevant—serosurvey	Naïve heifers and adult cows were serum-sampled to identify viruses responsible for the actual seroconversion in heifers and elucidate previous exposures of adult cows [26]. ELISA and VNT for the Simbu group and specific virus reactivity were used, respectively [26].
2011–2012	AKAV	A-H syndrome. Nervous-system signs in adult cattle and hypo-fertility in apparently healthy cattle	Specific AKAV RT-qPCR [5,23,25].
2014	AKAV AINO, SATV, SHAV	Not relevant—serosurvey	ELISA and VNT for the Simbu group and specific virus reactivity were used, respectively [26].
2014–2015 2018–2019	SHUV	A-H syndrome in cattle, sheep and goats	Cross-herd investigation, gathering clinical information from veterinary field practitioners across Israel. Virus isolation. Experimental challenge. [5,6,16].
2017–2018	PEAV	Blind newborn calf syndrome (initially not diagnosed) Detection in *C. imicola*, *C. oxystoma* and *C.* *puncticollis*	Pan Simbu group RT-qPCR (S,L segment) Specific PCR targeting PEAV segments (S, M, L) [17,18,26].
	SATV	Detection in C. imicola and C. oxystoma	PanSimbu group RT-qPCR(S, L) segments [18].
2018–2019	SBV	Samples originating from malformed ruminants. Detection in *C. imicola*, *C. oxystoma*, *C.* *puncticollis*, and *C. newsteadii*	Pan Simbu group nested PCR and qPCR [19]. Specific PCR targeting SBV segments (S, M, L). virus isolation. [19].

AKA, AINO, PEA, SAT, SHAV, SHU, SB—Akabane, Aino, Peaton, Satuperi, Shamunda, Shuni, Schmallenberg viruses.

## Data Availability

Not applicable.
